# Oral sensitization to whey proteins induces age- and sex-dependent behavioral abnormality and neuroinflammatory responses in a mouse model of food allergy: a potential role of mast cells

**DOI:** 10.1186/s12974-018-1146-0

**Published:** 2018-04-23

**Authors:** Danielle L. Germundson, Nicholas A. Smith, Lane P. Vendsel, Andrea V. Kelsch, Colin K. Combs, Kumi Nagamoto-Combs

**Affiliations:** 1 Department of Pathology, University of North Dakota School of Medicine and Health Sciences, 1301 North Columbia Road, Stop 9037, Grand Forks, ND 58202-9037 USA; 2 Department of Biomedical Sciences, School of Medicine and Health Sciences, University of North Dakota, 1301 North Columbia Road, Stop 9037, Grand Forks, ND 58202-9037 USA

**Keywords:** Behavior, Burrowing, Milk allergy, Hypersensitivity, Whey protein, Mast cells, Immunoglobulin, Microglia, Astrocytes, 5-hydroxymethylcytosine

## Abstract

**Background:**

Growing evidence has strengthened the association of food allergy with neuropsychiatric symptoms such as depression, anxiety, and autism. However, underlying mechanisms by which peripheral allergic responses lead to behavioral dysfunction are yet to be determined. Allergen-activated mast cells may serve as mediators by releasing histamine and other inflammatory factors that could adversely affect brain function. We hypothesized that eliciting food allergy in experimental animals would result in behavioral changes accompanied by mast cell accumulation in the brain. Our hypothesis was tested in a mouse model of milk allergy using bovine milk whey proteins (WP) as the allergen.

**Methods:**

Male and female C57BL/6 mice at 4 weeks (young) and 10 months (old) of age underwent 5-week WP sensitization with weekly intragastric administration of 20 mg WP and 10 μg cholera toxin as an adjuvant. Age-matched sham animals were given the vehicle containing only the adjuvant. All animals were orally challenged with 50 mg WP in week 6 and their intrinsic digging behavior was assessed the next day. Animals were sacrificed 3 days after the challenge, and WP-specific serum IgE, intestinal and brain mast cells, glial activation, and epigenetic DNA modification in the brain were examined.

**Results:**

WP-sensitized males showed significantly less digging activity than the sham males in both age groups while no apparent difference was observed in females. Mast cells and their activities were evident in the intestines in an age- and sex-dependent manner. Brain mast cells were predominantly located in the region between the lateral midbrain and medial hippocampus, and their number increased in the WP-sensitized young, but not old, male brains. Noticeable differences in for 5-hydroxymethylcytosine immunoreactivity were observed in WP mice of both age groups in the amygdala, suggesting epigenetic regulation. Increased microglial Iba1 immunoreactivity and perivascular astrocytes hypertrophy were also observed in the WP-sensitized old male mice.

**Conclusions:**

Our results demonstrated that food allergy induced behavioral abnormality, increases in the number of mast cells, epigenetic DNA modification in the brain, microgliosis, and astrocyte hypertrophy in a sex- and age-dependent manner, providing a potential mechanism by which peripheral allergic responses evoke behavioral dysfunction.

## Background

Food allergy has increasingly become prevalent worldwide [1] with a variety of symptoms including hives, respiratory and gastrointestinal manifestations, and even death by anaphylaxis. Although less recognized, food allergy has also been linked to abnormal psychosocial behavior and mood disorders, such as depression [2, 3], anxiety [3–6], attention-deficit hyperactivity disorder [2, 3, 6, 7], and autism [7–9]. These associations are mainly based on cohort studies, in which correlations between exacerbations of adverse behavior and consumption of suspected food were reported by patients and/or caregivers. However, the underlying mechanism by which food allergy contributes to the triggering and/or exacerbation of psychosocial symptoms is yet to be determined.

The development of a peripheral allergic reaction, or type I hypersensitivity, has been well-defined. During sensitization to an allergen, type 2 helper T-lymphocytes (Th2) facilitate the production of allergen-specific immunoglobulin E (IgE) by plasma cells, specifically differentiated B-lymphocytes [10, 11]. Mast cells and other immune mediator cells, in turn, become associated with IgE via Fcε receptors that are expressed on their cell surface. Finally, allergen recognition by the membrane-associated IgE leads to rapid degranulation of mast cells to release histamine, proteases, cytokines, and other inflammatory molecules into the circulation. These peripheral inflammatory molecules ultimately reach the brain and affect its cells and their functions, serving as periphery-derived neuroinflammatory mediators [12–14].

Alternatively, another mode of periphery-to-central communication may occur directly via mast cells in the brain. Because mast cells are present in the central nervous system (CNS) as resident cells or migratory cells from the periphery [15–17] and allergen sensitization can lead to accumulation of IgE in the brain [18], it may also be postulated that resident mast cells become activated and/or peripheral mast cells become recruited into the brain upon allergen exposure, releasing inflammatory molecules to affect brain function.

In this study, we therefore examined whether food allergen challenge would result in changes in the number of brain mast cells in sensitized mice. Since behavior manifestations by food sensitivity are often reported in younger populations, particularly in boys [19–23], we compared male and female mice at two age groups (“young,” 4 weeks old; “old,” 10 months old). We chose whey proteins (WP) from cow’s milk, one of the most common food allergens [24, 25], in the presence of cholera toxin (CT) to induce sensitization in mice [7, 26]. Sensitized mice were challenged with WP, and the presence of intact and degranulated mast cells in the brain was assessed in correlation with alteration in animal behavior and immune responses. Furthermore, 5-hydroxylmethylcytosine (5-hmC) immunoreactivity and glia cell morphology in the brain were histologically examined to demonstrate potential epigenetic DNA modifications and neuroinflammation as allergy-induced changes, respectively, in the central nervous system that may be associated with behavioral changes.

## Methods

### Materials

Flexible, 25-mm feeding needles for intragastric gavage were purchased from Instech Laboratories, Inc. (Plymouth Meeting, PA). Toluidine blue O dye was purchased from VWR International (Radnor, PA). ELISA reagents and normal goat serum were purchased from Thermo Fisher Scientific (Waltham, MA). The antibody against glial fibrillary acidic protein (GFAP) was obtained from Cell Signaling Technology Inc. (Danvers, MA). The rabbit anti-mast cell chymase antibody was purchased from Cloud-Clone Corp. (Katy, TX). The rabbit polyclonal antibody against mouse Iba1 was purchased from Wako Chemicals USA (Richmond, VA). The rabbit polyclonal antibody for 5-hmC was obtained from Active Motif (Carlsbad, CA). Vectastain Elite ABC HRP kits and VIP substrate were purchased from Vector Laboratories (Burlingame, CA). Spray-dried bovine milk whey protein, cholera toxin B subunit, and all other reagents were obtained from Sigma-Aldrich Co. (St. Louis, MO).

### Animals

C57BL/6 strain mice were bred and housed in the animal facility at the University of North Dakota (UND) with a 12-h light/dark cycle. The animals had access to food and water ad libitum. Four-weeks-old and 10-month-old male and female mice were randomly assigned to either sham or WP treatment groups (*n* = 5–8 per group). All animal use procedures were approved by the UND Institutional Animal Care and Use Committee.

### WP sensitization and challenge

**Fig. 1:**
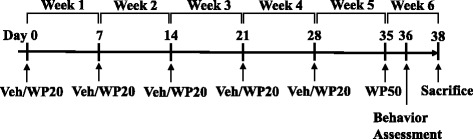
Timeline of the sensitization and behavioral assessment. At day 0, 4 weeks old or 10 months old male and female C57BL/6 mice were randomly assigned to either sham or WP sensitization groups (*n* = 5–8 per group). Starting with week 1, sham and WP mice received weekly intragastric administration of either 200 μL vehicle (PBS containing 10 μg cholera toxin as an adjuvant) or 20 mg WP in 200 μL vehicle for 5 weeks. At week 6, all animals were challenged with 50 mg WP in 200 μL PBS and their behavior was recorded 1 day after

Once a week for 5 weeks, the male and female mice in the WP treatment groups were intragastrically administered with 200 μl of phosphate-buffered saline (PBS) containing 20 mg WP with 10 μg CT as the adjuvant. The mice in the sham group received only the adjuvant in 200 μL PBS. At week 6, young animals, now 10 weeks old, and old animals 11.5 to 12 months old, were challenged with 50 mg WP in 200 μL of PBS. 1 day after the WP challenge, digging behavior of each animal was assessed as described below. A schematic for the sensitization and challenge timeline is depicted in Fig. 1.

### Digging behavior analysis

Cages with the dimensions of 38.7 cm (depth) × 24.8 cm (width) × 29.2 cm (height) were evenly filled with 5 cm of corncob bedding and were placed in opaque containers to prevent the animals from viewing adjacent cages. Mice were individually placed in the cages and were allowed to become accustomed to the new environment for 5 min. Their digging behavior was recorded for 10 min thereafter using ANY-maze software (Stoelting Co., Wood Dale, IL) and a CCD digital camera (C525 HD webcam, Logitech International, Newark, CA) placed above each cage. Since it has been reported that reusing the same bedding does not affect digging behavior in mice [27], after each recording, we simply scooped out approximately 1 cm of the bedding surface to remove any visible feces and replaced with fresh bedding. The inner walls of the cages were cleaned, and the bedding was leveled before placing another mouse. After recording the males, however, the cages were washed thoroughly and the entire bedding was replaced with fresh bedding before recording the females. Two examiners, who were blinded to the experimental condition of each animal, independently viewed the recordings and scored the presence (score = 1) or absence (score = 0) of digging behavior in each 10-s interval during the 10-min recording period (60 intervals total). The scores from the two examiners were averaged to determine the total number of the intervals during which animals exhibited digging behavior and were considered as the digging frequency. The inter-rater agreement was calculated as the percentage of the number of intervals agreed upon by both raters divided by the number of total (60) intervals [28].

### Serum and tissue sample collection

Animals were asphyxiated by CO_2_ inhalation 3 days after the WP challenge. Blood was collected after cardiac puncture and the remaining blood was cleared by intracardiac perfusion with sterile PBS. Sera were prepared by centrifuging the blood samples at 2000×*g* for 15 min at 4 °C after allowing clot formation for 30 min at room temperature. The brain from each mouse was hemisected longitudinally after removal. The right hemispheres were immediately frozen or stored in Allprotect solution (Qiagen Inc., Valencia, CA), while left hemispheres were immersion-fixed in 4% paraformaldehyde in PBS for 2 days at 4 °C. The ileum was divided into rostral and caudal sections and frozen-stored and immersion-fixed, respectively. The serum and frozen tissue samples were stored at − 80 °C until use.

### WP-specific IgE ELISA

Serum samples from the animals were analyzed for WP-specific IgE levels using enzyme-linked immunosorbent assay (ELISA). Each well of the 96-well microplate (Corning, Inc., Corning, NY) was coated with 20 μg/mL of WP solution in 100 mM sodium carbonate/bicarbonate buffer (pH 9.5) overnight at 4 °C. The wells were washed thoroughly in PBS containing 0.05% Tween-20 (PBST) and were incubated in PBST supplemented with fetal bovine serum (Assay Buffer, eBioscience ELISA Support Pack Plus, Thermo Fisher) for 2 h at room temperature. The serum samples were diluted 1:1 with the Assay Buffer before placing in the wells for 12–16 h incubation at 4 °C. The wells were washed thoroughly after the removal of the serum samples and incubated in anti-mouse IgE (eBioscience) at 1:1000 dilution followed by avidin-HRP solution (1:500 dilution) for 2 h at room temperature. After thorough rinses, TMB (3,3′,5,5′-Tetramethylbenzidine) substrate was added to each well and was incubated for 30 min at room temperature before the enzymatic reaction was terminated by the addition of 0.16 M sulfuric acid Stop Solution. The plate was immediately read at 450 nm using a BioTek ELx 800 microplate reader and Gen5 v3.02 software (BioTek Instruments, Inc., Winooski, VT).

### Staining and quantitation of mast cells

The fixed left brain tissues were embedded in a gelatin matrix and were sectioned at 40 μm as previously described [29], and the resulting floating sections were mounted on gelatin-coated glass slides and air-dried. The ileum was sectioned on a cryostat at 10 μm. The brain and ileum sections were immersed in freshly prepared 1% toluidine blue (TB) solution in 1% NaCl (pH 1.90) for 2 h or 30 min, respectively, in order to achieve metachromatic staining of mast cells. The presence of mast cells was observed using an Olympus BX-60 microscope and was photographed with a SPOT RT Slider CCD digital camera (Diagnostic Instruments, Inc., Sterling Heights, MI).

Four animals from the sham or WP-sensitized groups were randomly selected for the quantitation of brain mast cells. Every seventh section through the midbrain region, a total of 39 sections per young mouse and 26 sections per old mouse, was assessed for the presence of mast cells while differentiating granulated (intact metachromatically stained cells with granules confined within; Fig. 7b, top panel) from degranulated (presence of granules outside of the cells; Fig. 7b, bottom panel) mast cells. The localization of mast cells was recorded using the Allen Brain Atlas (http://www.brain-map.org/).

### Immunohistochemistry

Macrophages/microglia and astrocytes in the brain tissues were identified immunohistochemically using primary antibodies against Iba1 and GFAP, respectively. The paraformaldehyde-fixed left hemispheres were equilibrated with a 30% sucrose solution in PBS and were sectioned at 40 μm on a Leica SM2000R microtome. Brain sections were treated with 0.3% hydrogen peroxide and incubated in a blocking buffer (0.5% bovine serum albumin, 5% normal goat serum in PBS) to reduce endogenous peroxidase activity and non-specific staining. Both anti-Iba1 and anti-GFAP antibodies were used at 1:1000 dilution in the blocking buffer, and the sections were incubated for 12 h at 4 °C with gentle agitation. Immunoreactivity was visualized with the Vector VIP as the chromogen following the signal enhancement using Vector Elite ABC kit according to the manufacturer’s protocol. For the detection of epigenetic DNA modification, brain tissues first underwent heat-induced epitope retrieval for 30 min at 37 °C in 1 M hydrochloric acid and were then immunostained with an anti-5-hmC antibody (1:8000) as described above. Sections were thoroughly rinsed in PBS and mounted on gelatin-coated glass slides. Air-dried slides were dehydrated through a series of increasing concentrations of ethanol solutions, were de-fatted in Histo-Clear (National Diagnostics, Atlanta, GA), and were coverslipped in Permount mounting medium (Fisher Scientific, Hampton, NH). The specimens were observed and photographed as described above. The paraformaldehyde-fixed ileum samples were also equilibrated with a 30% sucrose solution in PBS, were sectioned at 10 μm on a Leica CM1850 cryostat, and were immediately mounted on subbed glass slides. Primary antibodies against CD68 and mast cell chymase 1 (CMA1) were used at 1:1000 and 1:200 dilution, respectively, to stain the intestinal sections.

### Densitometric analyses of immunohistochemical staining

Immunohistochemical staining of the brain tissues was quantified as previously described [30]. Briefly, photomicrographs of the regions of interest were taken using a × 4 objective on an Olympus BX60 microscope and a SPOT RT Slider digital camera (*n* = 5–6). Exposure settings were kept consistent within age groups for comparison. Each image was inverted, and the background was standardized by setting it to black using Adobe Photoshop CS6 software. Indicated brain regions were defined, and the optical density (OD) within the selected region of each image was calculated by dividing the mean gray value by the defined area.

### Reverse transcriptase-quantitative PCR (RT-qPCR)

Total cellular RNA was isolated from ileum samples using TRIzol reagent (Thermo Fisher Scientific) according to the manufacturer’s protocol and quantitated using a NanoDrop Spectrophotometer (Thermo Fisher Scientific). For the detection of occludin, ileum samples were gently lysed with zirconium oxide beads (0.5 mm dia.) at setting 3 for 3 min at 4 °C in a Bullet Blender tissue homogenizer (Next Advance, Inc., Averill Park, NY), leaving muscular layers and connective tissue intact. For the detection of tryptase (*Tpsab1*), RNA was isolated from tissue samples that were fully homogenized to include the serosal layer, within which mast cells were primarily found in our histological samples. Reverse-transcriptase reactions were carried out using 1 μg of RNA and iScript cDNA Synthesis Kit (Bio-Rad Laboratories, Hercules, CA) on an Eppendorf Mastercycler Nexus Gradient thermal cycler (Eppendorf, Hauppauge, NY). Target expressions were determined from the resulting cDNA by qPCR assays using 100 ng of the DNA template, iTaq Universal SYBR Green Supermix, and target-specific primer pairs (MilliporeSigma, St. Louis, MO) on a CFX98 C1000 Thermal cycler (Bio-rad). The expression of *Gapdh* was detected for each sample using a specific primer set (qMmuCED0027497, PrimePCR SYBR Green Assay, Bio-Rad) and used a reference gene.

Amplification reactions were performed with 40 cycles of denaturation (5 s at 95 °C) and annealing/extension (30 s at 60 °C) followed by a melt-curve analysis. Data was acquired and analyzed with CFX Manager 3.1 software (Bio-Rad). Target Cq values for each sample were normalized to corresponding reference Cq value to obtain ΔCq value for the calculation of the target gene expressions (2^−ΔCq^) and were shown as the fold change (ΔΔCq) compared to the expression of the sex- and age-matched sham controls. For the target primer sequences, see Table 1.

**Table 1:** Sequences of the primers used in RT-qPCR

Target	Symbol	Forward/reverse primer sequences	
Occludin	*Ocln*	F	5′-AAAGCAAGTTAAGGGATCTG-3′
		R	5′-TGGCATCTCTCTAAGGTTTC-3′
Tryptase	*Tpsab1*	F	5′-GCCAATGACACCTACTGGATG-3′
		R	5′-GAGCTGTACTCTGACCTTGTTG-3′

### Statistical analysis

Differences in digging behavior between the sexes and the treatment groups were statistically compared by performing two-way ANOVA followed by Fisher’s LSD test using GraphPad Prism 7 software (GraphPad Software, Inc., San Diego, CA). A *p* value of less than 0.05 was considered statistically significant.

## Results

### WP-sensitized male mice exhibited decreased digging activity following an oral antigen challenge

**Fig. 2:**
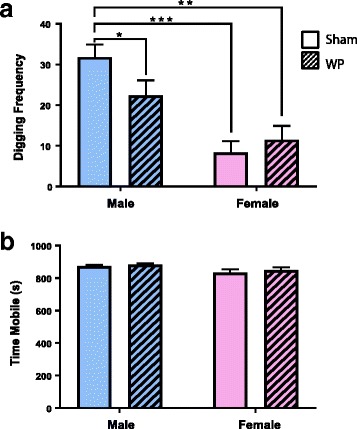
Digging frequency and overall activity of young male mice after an antigen challenge. Two scorers, to whom the treatment condition of each mouse was undisclosed, independently observed the videos recorded during the digging behavior assessments. The presence (1 point) or absence (0 point) of digging was scored for each of the 10 s intervals during the test period (10 min). The points scored by the two scorers were averaged for each mouse and were used as the mouse digging frequency. **a** The digging frequency for each group is presented as the group average ± standard error. **b** Total mobile time in seconds was computed by ANY-maze software to assess general immobility in the mice. The open bars and hashed bars indicate sham and WP-sensitized groups, respectively. Male: *n* = 8; female *n* = 5-6, *p < 0.05, **p < 0.0005, ***p < 0.0001

**Fig. 3:**
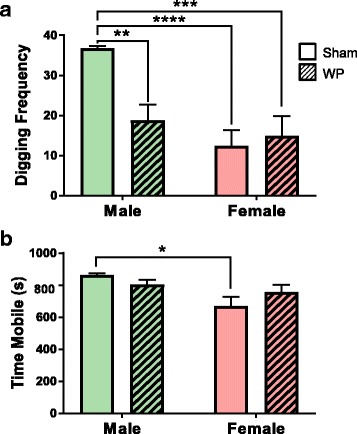
Digging frequency and overall activity of old male mice after an antigen challenge. Two scorers, to whom the treatment condition of each mouse was undisclosed, independently observed the videos recorded during the digging behavior assessments. The presence (1 point) or absence (0 point) of digging was scored for each of the 10 s intervals during the test period (10 min). The points scored by the two scorers were averaged for each mouse and were used as the mouse digging frequency. **a** The digging frequency for each group is presented as the group average ± standard error. **b** Total mobile time in seconds was computed by ANY-maze software to assess general immobility in the mice. The open bars and hashed bars indicate sham and WP-sensitized groups, respectively. Male, *n* = 6; female, *n* = 7, *p < 0.05, **p < 0.005, ***p < 0.001, ****p<0.0005

Digging activity is thought to reflect rodent burrowing behavior [27, 31]. To test whether this instinctive behavior would be affected by WP-sensitization, all animals were orally challenged with 50 mg of WP at week 6, and their digging activity was observed the following day. When placed in a new cage filled with fresh, thick bedding material, the male mice, in general, exhibited more robust digging activity compared to female mice (Fig. 2a, *p* < 0.0001; Fig. 3a, *p* < 0.0005). Interestingly, the digging frequency of WP-sensitized male mice was decreased by approximately 30% in young mice (sham 32 ± 3, *n* = 8; WP 22 ± 3, *n* = 8; *p* < 0.05) and by 50% in old mice (sham 36.7 ± 0.6, *n* = 6; WP 19 ± 4, *n* = 6; *p* < 0.005), indicating that the WP sensitization reduced their instinctive burrowing behavior. In contrast, the effect of WP sensitization on this behavior was not apparent in female mice of both age groups, and sham and WP-sensitized young female groups displayed 25 and 38% (sham 8 ± 3, *p* < 0.0001; WP 12 ± 3, *p* < 0.0005), and sham and WP-sensitized old female groups showed 32 and 39% (sham 12 ± 4, *p* < 0.0005; WP 15 ± 5, *p* < 0.001) digging frequency of their age-matched sham males. In order to ensure that the decreased digging activity observed in WP-sensitized male mice was not due to lethargy, overall activity levels during the test period were also assessed. A comparison of total mobile time during the recording period indicated that the four groups of young mice were equally active (Fig. 2b). While the older female sham mice showed a slight but significantly lower level of overall activity (Fig. 3b) compared to male sham mice, this difference did not affect the similarity in the digging activity levels between the two female groups (Fig. 3a). This result showed that WP sensitization decreased digging behavior in WP-challenged mice in a sex-dependent manner, without affecting the total level of mobility.

### The level of WP-specific IgE levels increased in the WP-sensitized mice in an age- and sex-dependent manner

**Fig. 4:**
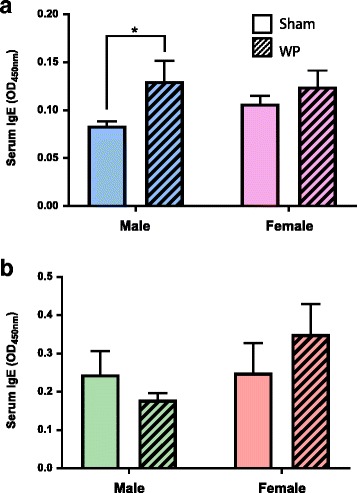
Assessment of WP-specific IgE levels in the sera from sham and WP-sensitized mice using ELISA. Relative levels of WP-specific IgE were determined in the sera from sham or WP-sensitized young (**a**) and old (**b**) male and female mice. Each serum sample was diluted 1:1 with assay buffer prior to the assay. The amounts of WP-specific IgE were determined by the colorimetric substrate reaction and the average optical density (OD) at 450 nm for the experimental groups were compared (average OD ± standard error). The open bars and hashed bars indicate sham and WP-sensitized groups, respectively. Young male, *n* = 7–8; young female, *n* = 8; old male, *n* = 6; old female, *n* = 7, *p < 0.05

To determine whether the WP-sensitization protocol indeed induced immunoglobulin-mediated hypersensitivity to the antigen, the serum level of WP-specific IgE was measured using ELISA. For both young (Fig. 4a) and old (Fig. 4b) groups, the serum IgE levels did not significantly differ between male and female sham groups (Fig. 4, open bars). When these values were compared with their respective WP-sensitized groups, however, the level of IgE was found to be elevated in the young WP-sensitized male group by approximately 40% (sham 0.084 ± 0.005, *n* = 7; WP 0.13 ± 0.02, *n* = 8, *p* < 0.05). In contrast, we did not observe significant increases in the serum IgE levels in the older males or females of either age group. These data indicated that the WP-sensitization protocol moderately induced IgE-mediated hypersensitivity in young male mice, and their decreased digging behavior correlated with the higher levels of serum IgE. The lack of IgE elevation in the old WP-sensitized male mice that exhibited a substantial decrease in digging behavior suggests that the sensitization protocol might have elicited alternative immune responses other than IgE-mediated hypersensitivity.

### WP sensitization elicits changes associated with mast cell functions in the ileum

**Fig. 5:**
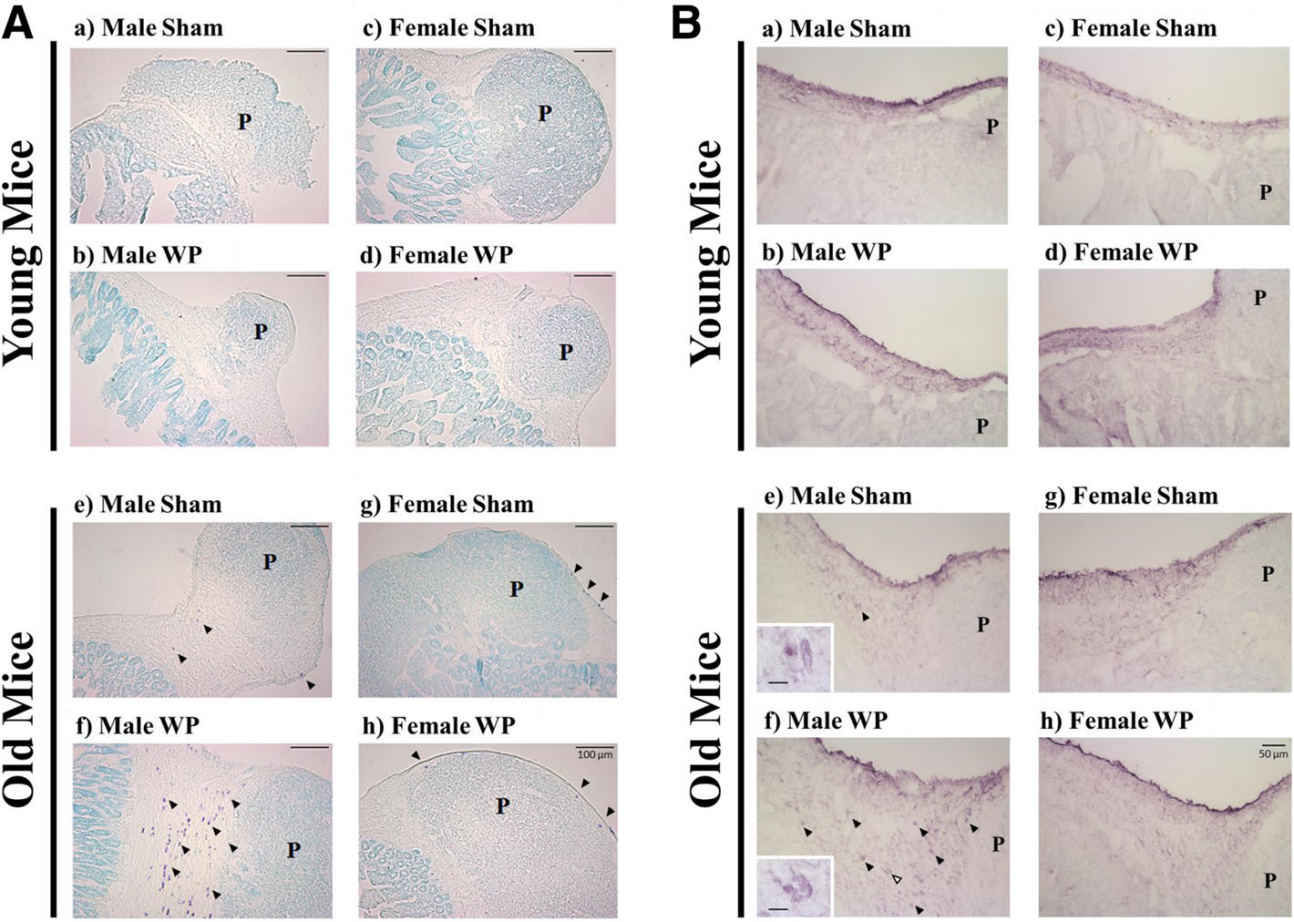
Histological evaluation of the ileal tissues from sham and WP-sensitized young and old mice. Paraformaldehyde-fixed frozen ileal tissues adjacent to Peyer’s patches (labeled with a ‘**P**’) were cryosectioned at 10 μm. Mast cells were detected as metachromatically stained dark purple cells with TB staining (**A**) or light purple cells in CMA1 immunohistochemical staining (**B**). The location and number of the stained cells were qualitatively assessed in sham (**a**, **c**, **e**, **g)** and WP-sensitized (**b**, **d**, **f**, **h**) mice. Representative images were taken using a ×10 objective (**A**, scale bar = 100 μm), or a ×20 objective (**B**, scale bar = 50 μm)

To assess the presence of mast cells in the intestines where they might be positioned for rapid inflammatory responses to food-allergen exposure, the ileums of the sham and WP-sensitized mice were stained with acidic TB. Although we did not detect metachromatically stained mast cells in the ileums from the young mice (Fig. 5A, a–d), we observed dark purple cells in the serosal layer and submucosa of the old mouse ileum sections (Fig. 5A, e–h arrowheads). A greater number of TB-stained mast cells were found in the old WP-sensitized male mice (Fig. 5A, f). To validate the TB staining, mast cells in the ileums were also detected immunohistochemically for CMA1 (Fig. 5B). While staining controls without CMA1 primary antibody showed minimal background staining (not shown), a number of immunoreactive cells were found in ileal submucosa and serosa of all animals (Fig. 5B). These cells appeared morphologically distinct from TB-stained mast cells and lacked a distinct granular appearance. However, some CMA1-immunoreactive cells that are readily identifiable as mast cells morphologically were observed in the ileums of old male mice (see insets in Fig. 5B, e, and f, arrowheads). The distribution of these cells was similar to TB stained cells and more abundant in old WP-sensitized males, corroborating the observations we made from the TB staining. These results demonstrated that mast cells are present in the gut and suggested that allergen entry to this area could trigger robust responses via degranulation of these cells.

**Fig. 6:**
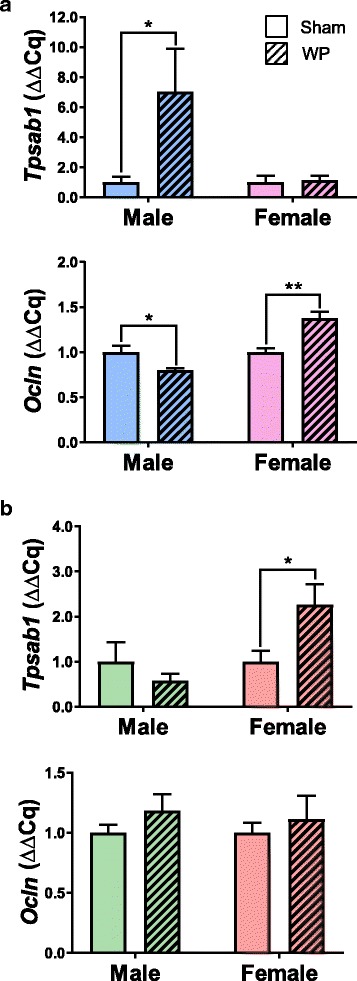
Expression of tryptase and occludin in the ileums of young and old mice. The levels of tryptase (*Tpsab1*) and occludin (*Ocln*) expression were determined in the total ileum RNA samples isolated from sham or WP-sensitized young (**a**) and old **(b**) male and female mice using RT-qPCR. The values indicate average Cq expression (2^−ΔCq^) ± standard error for each group. The open bars and hashed bars indicate sham and WP-sensitized groups, respectively. Young male, *n* = 7; young female, *n* = 5–6; old male, *n* = 6; old female, *n* = 5–6, *p < 0.05, **p < 0.001

To further assess WP-sensitization-mediated changes in the ileum that are associated with mast cell functions, we determined the expression levels of tryptase and occludin. Tryptase is a protease released from activated mast cells and has been shown to decrease the level of occludin in intestinal epithelial cells [32]. RT-qPCR results showed that the expression of tryptase (*Tpsab1*) was elevated by 7-fold in young WP-sensitized male mice when compared to the age- and sex-matched sham mice (*p* < 0.05) while this WP-mediated effect was not observed in female mice (Fig. 6a). As expected from the increased *Tpsab1* expression observed in young male WP-sensitized mice, occludin expression (*Ocln*) in these mice was decreased by approximately 20% (0.79 ± 0.02 fold change, *p* < 0.05). Interestingly, we observed a contrasting effect of WP sensitization on *Ocln* expression in young WP-sensitized female mice. Their *Ocln* levels were slightly but significantly increased (1.38 ± 0.07 fold change, *p* < 0.001), suggesting that another regulatory pathway for *Ocln* expression may exist. In older mice, WP sensitization increased the expression of *Tpsab1* in female mice but not in male mice, even though TB stained mast cells were more readily found in the ileum sections from the latter group (Fig. 6b). WP sensitization did not affect the expression of *Ocln* in the ileum of old mice. These results indicated that WP sensitization resulted in changes associated with mast cells and their functions in a complex, age- and sex-specific manner.

### Brain mast cells were relatively localized to the midbrain regions, and their number was increased in the young WP-sensitized animals

**Fig. 7:**
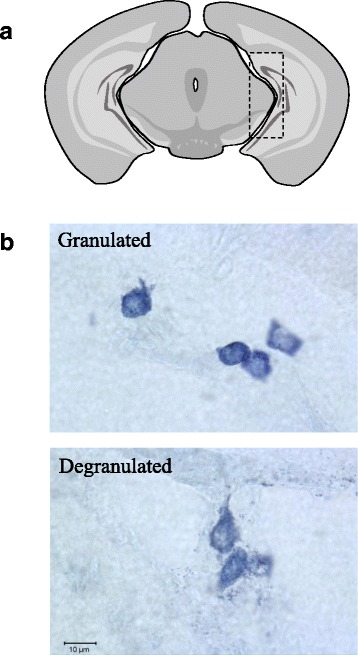
Mast cells in the brain. **a** A schematic diagram of a coronal mouse brain section through the midbrain, where the majority of the brain mast cells were localized (dotted rectangle). **b** Examples of granulated (top) and degranulated (bottom) mast cells found in the mouse brain

**Fig. 8:**
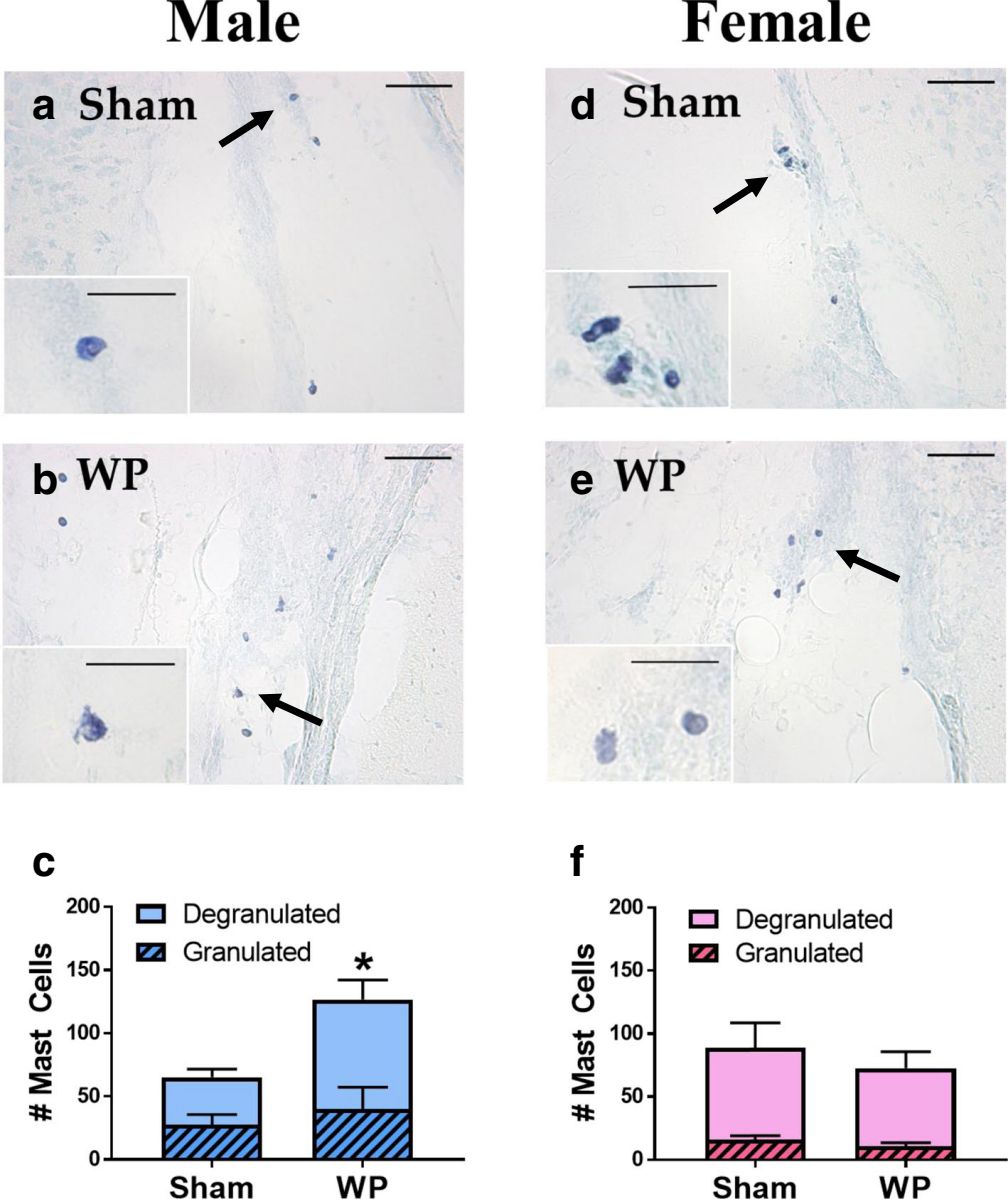
TB staining and quantitation of mast cells in the young mouse brains. For the photomicrographs **a**, **b**, **d**, and **e**, paraformaldehyde-fixed brains from young sham (**a**, **d**) and WP-sensitized (**b**, **e**) male (**a**, **b**) and female (**d**, **e**) mice were coronally frozen-sectioned at 40 μm and stained with TB. The lower left insets in the panels **a**, **b**, **d**, and **e** show the cells indicated with the arrows at a higher magnification. The images in the panels were taken with a ×20 objective (scale bar = 50 μm), whereas the insets were taken with a ×40 objective (scale bar = 20 μm). Quantitative comparisons show the number of granulated (hashed bars) and degranulated (open bars) mast cells observed in male (**c**) and female (**f**) brain sections. Values indicate group average ± standard error (*n* = 4), *p < 0.05

**Fig. 9:**
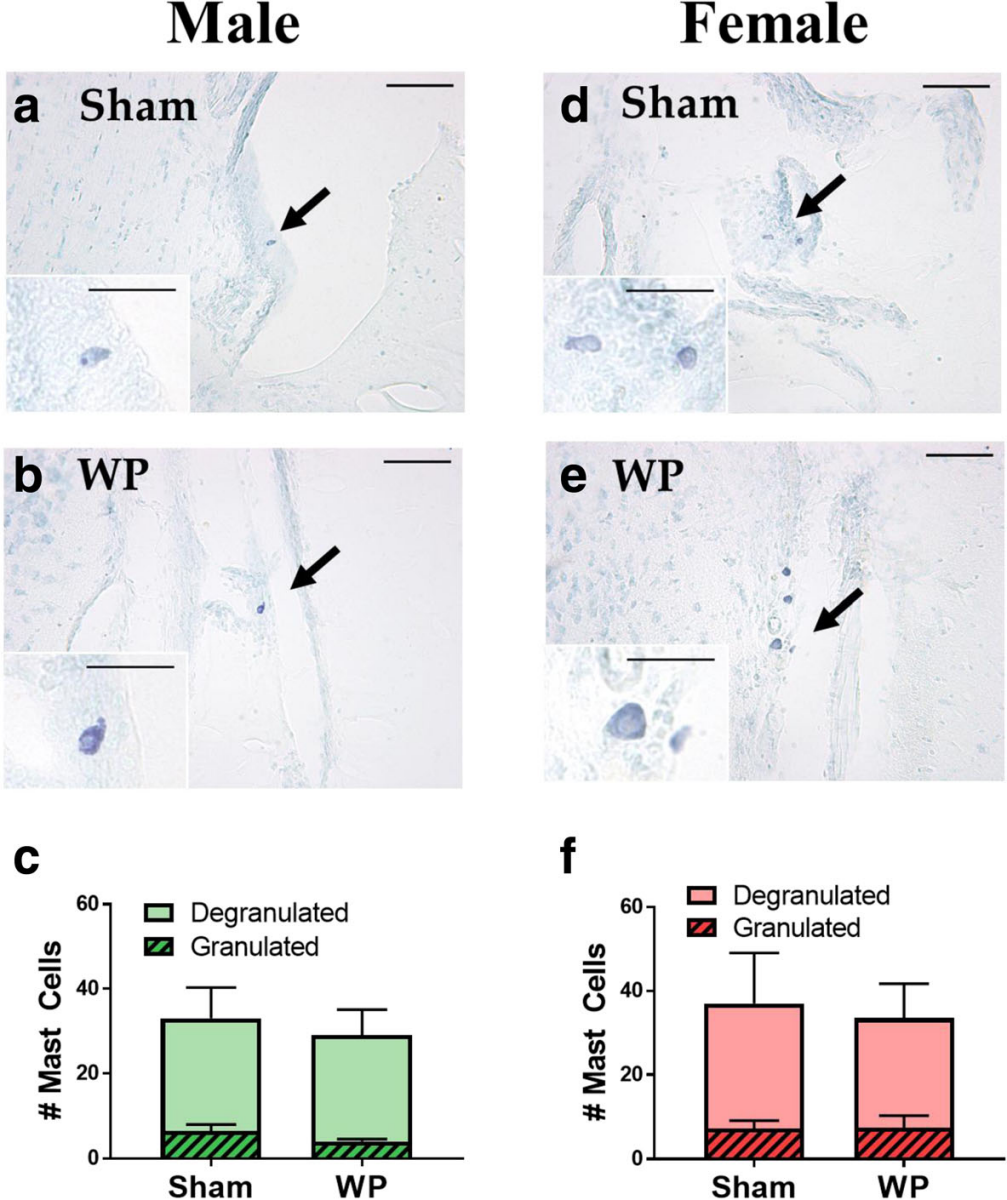
TB staining and quantitation of mast cells in the old mouse brains. For the photomicrographs **a**, **b**, **d**, and **e**, paraformaldehyde-fixed brains from old sham (**a**, **d**) and WP-sensitized (**b**, **e**) male (**a**, **b**) and female (**d**, **e**) mice were coronally frozen-sectioned at 40 μm and stained with TB. The lower left insets in the panels **a**, **b**, **d**, and **e** show the cells indicated with the arrows at a higher magnification. The images in the panels were taken with a ×20 objective (scale bar = 50 μm), whereas the insets were taken with a ×40 objective (scale bar = 20 μm). Quantitative comparisons show the number of granulated (hashed bars) and degranulated (open bars) mast cells observed in male (**c**) and female (**f**) brain sections. Values indicate group average ± standard error (*n* = 4)

In order to determine whether the number of mast cells in the brain would be affected by WP sensitization, brain sections from the sham and WP-sensitized animals were stained with acidic TB, and the presence and distribution of mast cells were examined. Mast cells were sporadically found in the brain, and many of the sections displayed only a few or no mast cells. However, in midbrain-level sections, clusters of dark purple, metachromatically stained mast cells were observed within the area between the lateral midbrain and medial hippocampus in both age groups (Fig 7a, dotted rectangle; Figs. 8 and 9). Other areas in which mast cells were observed included, but were not limited to, the striatum, thalamus, habenula, hippocampus, cerebral white matter, and various cortical areas. Both granulated and degranulated mast cells were present in the brains from all animals (Figs. 8 and 9). This observation was not unexpected since it has been reported, at least in the rat, that degranulation of mast cells is a normal physiological phenomenon affected by reproductive and stress hormones [17, 33, 34] and brain mast cells contribute approximately one half of the histamine in the brain [33]. For quantitative comparisons between the sham and WP-sensitized mice, mast cells in the brain sections were counted, differentiating granulated and degranulated forms based on their morphology (Fig. 7b). The quantitation of mast cell numbers indicated that approximately 2-fold more degranulated mast cells were present in young WP-sensitized male mouse brains than the corresponding shams while the number of granulated mast cells did not differ significantly (Fig. 8c). This WP-sensitization-dependent variation in the mast cell numbers was not observed in young female mice (Fig. 8f) or older mice of either sex (Fig. 9c and f). These results suggested that WP-sensitization increased the number of brain mast cells only in young male mice, and the majority of the mast cells had been activated, based on their degranulated morphology.

### Modest differences in the patterns of 5-hydroxymethylated DNA staining were observed in WP-sensitized mouse brains

**Fig. 10:**
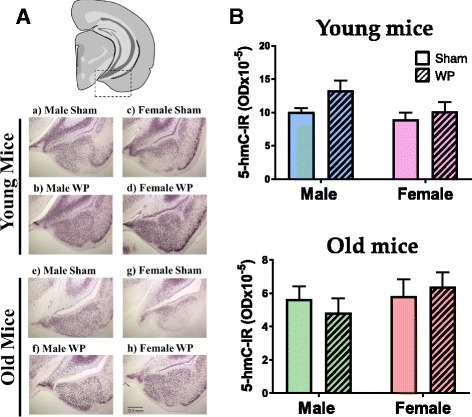
Detection of epigenetic DNA modifications with 5-hmC immunohistochemistry. **A** Paraformaldehyde-fixed brains from young (a–d) and old (e–h) brain tissues were coronally frozen-sectioned at 40 μm and were stained with an anti-5-hmC antibody. Immunoreactivity in the area including the temporal lobe and amygdala (dotted rectangle in the top diagram) was qualitatively evaluated in sham (a, c, e, g) and WP-sensitized (b, d, f, h) mice. Representative photomicrographs were taken using a ×4 objective (scale bar = 0.5 mm). **B** Immunoreactivity to 5-hmC (5-hmC-IR) within the young and old mouse brains was quantified by densitometric analyses of the digital photomicrographs taken with a ×4 objective. The values indicate group average optical density ± standard error (*n* = 5–6). Top graph, young mice; bottom graph, old mice

To continue examining whether allergen-mediated peripheral inflammation occurring in the intestines could propagate effects in the brain, we hypothesized that WP sensitization would lead to epigenetically modified gene expression. To test this idea, brain sections from sham and WP-sensitized mice were immunostained for 5-hmC (Fig. 10A). Many regions of the brains displayed intense immunoreactivity localized to nuclei. For example, a robust increase in 5-hmC immunoreactivity was observed in the temporal lobe and amygdala regions of WP-sensitized male and female mice at both ages (Fig. 10A). The qualitative observations of epigenetic DNA modifications seemed to provide the best correlate of WP sensitization across age and sex. However, quantitation of the immunostaining did not fully support this observation (Fig. 10B). Although there was an upward trend in 5-hmC immunoreactivity in WP-sensitized mice, particularly in young male mice, the difference did not reach a statistical significance (sham 10.1 ± 0.6 × 10^− 5^; WP 13 ± 1 × 10^− 5^; *p* = 0.06). The trend was not observed in WP-sensitized old male mice. Thus, while our qualitative comparisons suggested differences in 5-hmC immunostaining in the brain, densitometric quantitation of 5-hmC staining provided limited support. To substantiate our observation, further quantitative analysis is required.

### WP-sensitization altered perivascular astrocyte morphology in the old male brain

**Fig. 11:**
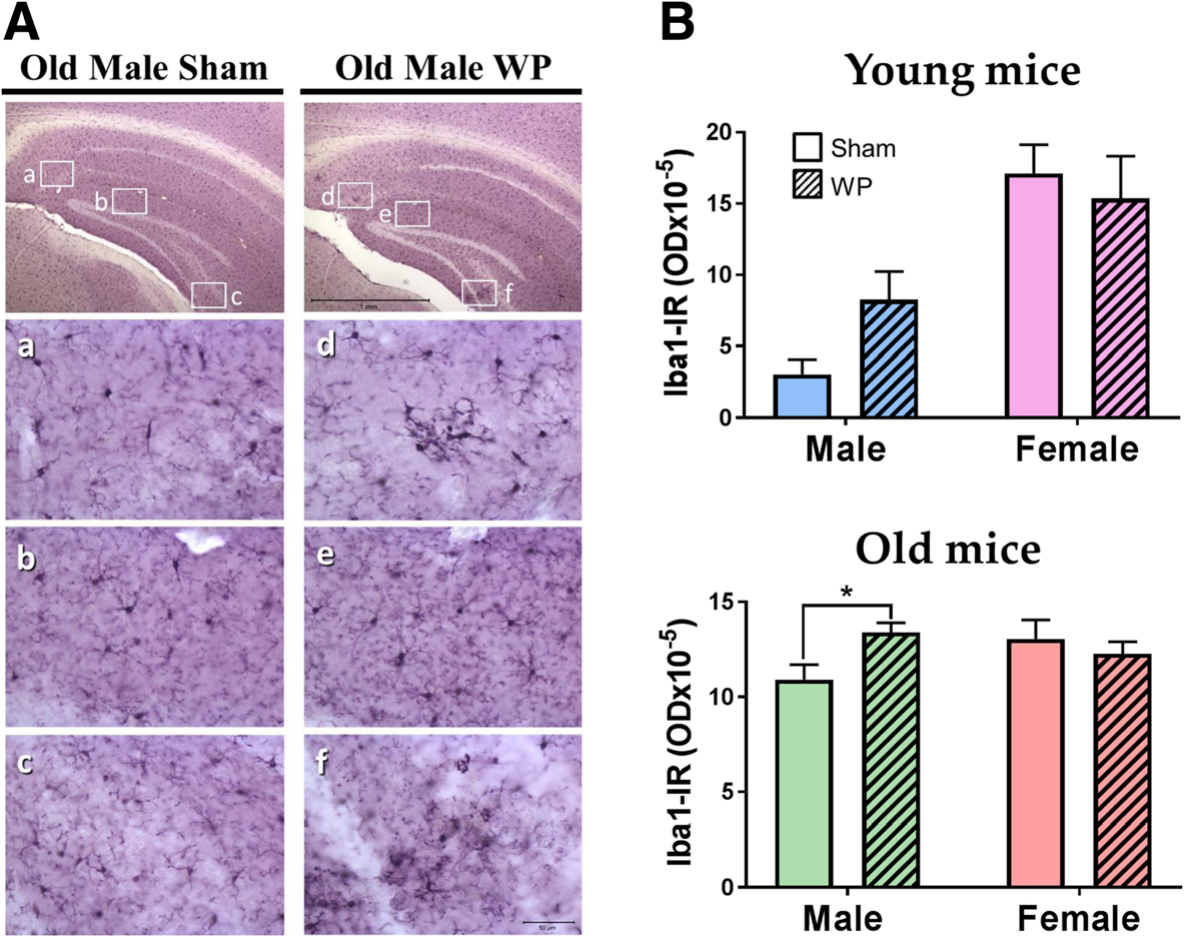
The effect of WP-sensitization on Iba1 immunoreactive microglia in the old mouse brains. **A** Microglia were identified using anti-Iba1 immunohistochemical staining in the brain sections (40 μm) of the old sham and WP-sensitized mice. Subtle differences between sham and WP-sensitization in Iba1 immunoreactivity were noted in the hippocampal region of the old mice. (top panels) Representative photomicrographs were taken using a ×4 objective (scale bar = 1 mm). The rectangles within indicates where the high-power photomicrographs **a–f** were taken using a × 40 objective. **B** Immunoreactivity to Iba1 (Iba1-IR) within the young and old mouse brains was quantified by densitometric analyses of the digital photomicrographs taken with a ×4 objective. The values indicate group average optical density ± standard error (*n* = 5–6), *p < 0.05. Top graph, young mice; bottom graph, old mice

We next assessed whether WP-sensitization would affect glial cell activation phenotype. Microglia and astrocyte reactivity were visualized by immunohistochemical staining against Iba1 and GFAP, respectively. Iba1 immunoreactivity was found throughout the brain in all animals. Although the staining patterns between the sham and WP-sensitized mice were not strikingly different, subtle differences were apparent in the hippocampal regions, with WP-sensitized male mice having more immunoreactive cells than the sham or female groups within the respective age groups (Fig. 11A). Quantification of the staining by densitometric analyses of the brain sections validated this observation in the old mouse groups and indicated that Iba1 immunoreactivity was significantly increased in WP-sensitized male mice (Fig. 11B, bottom, sham 10.9 ± 0.8 × 10^− 5^; WP 13.4 ± 0.5 × 10^− 5^, *p* < 0.05). A similar increase was also observed in young WP-sensitized male mice, though the difference did not reach statistical significance (Fig. 11B, top, sham = 3 ± 1 × 10^− 5^; WP 8 ± 2 × 10^− 5^; *p* = 0.075).

**Fig. 12:**
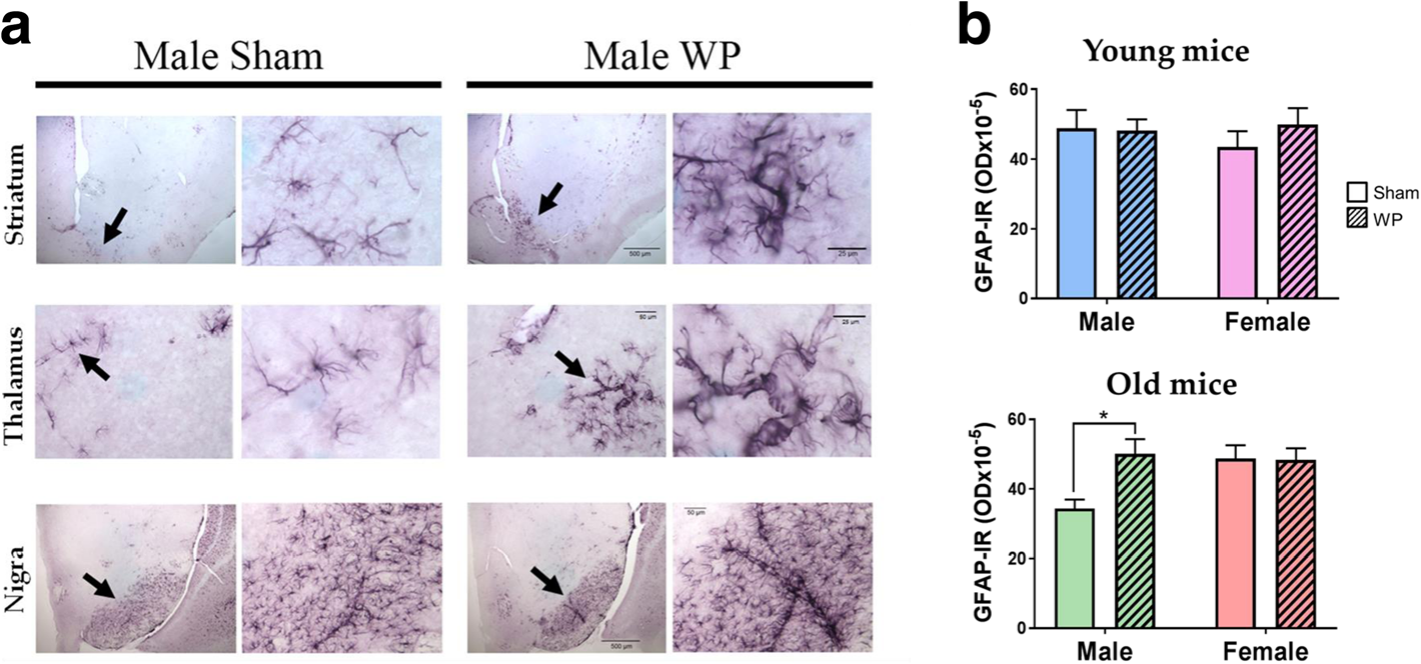
The effect of WP-sensitization on GFAP-immunoreactive perivascular astrocytes in the old male mouse brains. **a** Astrocytes were identified using GFAP-immunohistochemical staining in the brain sections (40 μm) of the old sham and WP-sensitized male mice. The striatum, thalamus, and substantia nigra are shown as low-power (left panels in each of the Sham and WP columns) and high-power (right panels) images. The arrows in the left panels indicate the areas where the high-power images were taken. Scale bar sizes are as indicated. Representative images are shown (*n* = 6). **b** Immunoreactivity to GFAP (GFAP-IR) within the young and old mouse brains was quantified by densitometric analyses of the digital photomicrographs taken with a ×4 objective. The values indicate group average optical density ± standard error (*n* = 5–6), *p < 0.005. Top graph, young mice; bottom graph, old mice

Furthermore, we observed noticeable hypertrophy of perivascular astrocytes in select regions of the old WP-sensitized mouse brain (Fig. 12a). Blood vessels within the ventral striatum, thalamus, and substantia nigra were densely walled with thick astrocytic processes suggesting alterations in brain vascular properties. There were no salient differences between the GFAP-stained cells in the sham and WP-sensitized groups of younger animals (not shown). Densitometric quantification revealed that GFAP immunoreactivity was increased approximately by 30% in WP-sensitized old male mice (Fig. 12b, bottom). These results indicated that WP-sensitization notably affected the perivascular astrocytes and this effect may be age- and sex-dependent.

## Discussion

The goal of this study was to establish concrete evidence that oral sensitization of mice to a food allergen causes changes in their normal behavior and brain physiology. Contributory roles of food allergy in behavioral abnormality have been suggested in clinical studies [2, 3, 6, 35, 36]. However, it is difficult to determine from these studies whether the peripheral inflammation triggered by allergic responses alone is sufficient to elicit behavioral changes in humans because their behavior may be influenced by fear of allergen exposure [5, 6] or negative social interactions with bullying peers [37, 38]. The use of a mouse model excludes these additional psychosocial factors that are unique to humans who are aware of their conditions and dissects out behavioral changes triggered by allergy-induced peripheral immune responses. We demonstrated that weekly treatment with WP in the presence of an adjuvant for 5 weeks resulted in abnormal burrowing behavior in male mice without affecting their overall activity levels. Thus, experimental WP-sensitization of otherwise healthy mice caused deviations in their instinctive digging behavior, at least in males, indicating that food allergy independently triggers behavior alteration.

In our study, we chose the C57BL/6 mouse strain for our food allergy model although BALB/c and C3H/HeJ are more commonly used for immunological studies. Our rationale for not using these latter strains was that these mice have been reported to have greater tendencies to exhibit severe anaphylactic reactions often resulting in hypothermia, breathing difficulty, immobility, and death after sensitization [39–42]. On the other hand, C57BL/6 mice did not show any obvious signs of anaphylaxis in our study, allowing us to examine behavioral deviations after food challenge. Strain-dependent differences in immune responses have been reported, and C57BL/6 mice are known to have moderate responses to allergen sensitization compared to other strains [42, 43]. Although there are limitations in any animal model when comparing to human diseases, the modest responses observed in C57BL/6 mice may reflect circumstances where non-anaphylactic hypersensitive patients continue to consume offensive food. In such patients, chronic allergen exposures may cause low-grade inflammation, which has been linked to behavioral disorders [44].

For the behavioral assessments of our mouse model, we observed digging activity, which represents stereotypical burrowing behavior of mice. Digging, assessed directly via actions of bedding displacement or indirectly via marble burying, is also thought to reflect repetitive, compulsive-like and/or anxious behavior often observed in autistic patients [45–48]. In mouse models of autism spectrum disorder, different strains with mutations of various autism-related genes show either an increase or decrease in digging/marble burying behavior [46]. In our mouse model of food allergy-induced behavioral disorder, we assessed digging activity as an instinctive behavior that might be noticeably affected with allergen challenge. In general, we observed that male mice exhibited approximately 3-fold higher digging activity than age-matched females (Figs. 2a and 3a, open bars). Although the older female mice showed a slight but significantly lower level of overall activity compared to their male counterparts (Fig 3b), it did not negate the difference in the digging frequency between the male and female sham groups. Perhaps more importantly, WP-sensitization decreased the burrowing behavior in male mice of both age groups but not in female mice (Figs. 2a and 3a). The absence of the post-sensitization effect on female behavior may be explained by their low basal burrowing activity. Alternatively, the results may demonstrate that male mice are more susceptible to WP-sensitization and/or changes in their behavior phenotype. In support of this notion, it is reported in human studies that males are more afflicted by IgE-mediated food allergy than females in younger populations [49–52], although the trend becomes reversed in older populations [49]. Our results from WP-specific IgE ELISA corroborate these sex- and age-dependent phenomena (Fig. 4). In addition, behavioral disorders such as autism spectrum disorder [53], attention-deficit hyperactivity disorder (ADHD) [20, 21, 23], and obsessive-compulsive disorder [19, 22, 54] are more prevalent in males, and the symptoms of girls diagnosed with ADHD are more implicit and less noticeable than boys [20, 21]. Thus, the sex difference observed in our experimental mice is consistent with the findings in human patients afflicted independently with allergy or behavioral disorders. Although this sex dichotomy in the susceptibility to allergy and behavioral disorders requires further investigation, it likely stems from fundamental differences in hormonal and immune cell compositions between male and female mice. Indeed, importance of such sex differences has been emphasized in mechanical and inflammatory pain paradigms [55]. Interestingly, resistance to physiological changes in female animals to experimental manipulations has also been reported in studies investigating stress-induced behavioral and neuronal alterations [56]. Nonetheless, our results indicate that male C57BL/6 mice are more susceptible to WP-sensitization and the effect of the antigen manifests as a decrease in their stereotypical burrowing behavior.

As briefly mentioned above, WP-specific IgE levels in young male mice (Fig. 4a) seemed to show an inverse correlation with digging frequency. However, the sera from old male mice (Fig. 4b) did not present significant changes in the IgE levels even though the behavioral change, when compared to their age- and sex-matched sham group, was most evident in this group (Fig. 3a). This discrepancy may be explained by the finding that detection of allergen-specific IgE ELISA can be obscured by high levels of allergen-specific IgG in mouse samples [57]. It should also be noted that we were not able to generate absolute values for the amount of WP-specific IgE in the serum samples. Instead, our ELISA data represents relative amounts of WP-specific IgE indicated in optical density (OD). Background signals from the assay might have contributed to the discrepancy. Alternatively, it is possible that WP elicits inflammatory responses independently of IgE production in the older mice. Such IgE-independent immune responses to food have been described as non-IgE-mediated food allergy or food hypersensitivity [58]. Interestingly, behavioral deviations have also been reported in patients with non-IgE-mediated food allergy [59].

In non-IgE-mediated food allergy, infiltration of eosinophils [59] and mast cells [60] have been found in gastrointestinal tissues. We observed increased numbers of metachromatically stained mast cells in the ileums from the old WP-sensitized male mice but not in age-matched female or young mouse groups (Fig. 5a). This result was further confirmed by mast cell chymase immunohistochemical staining (Fig. 5b). Our histological observations, combined with the WP-specific IgE results, may indicate that the non-IgE-mediated mechanism plays a key role in the old male mice while the IgE-mediated mechanism, at least in part, is responsible for producing allergic responses in the young male mice. More extensive analyses of mast cells and other leukocyte infiltrations in the intestinal tissues are warranted in our future studies.

To demonstrate the biological events associated with mast cell functions in the intestines of the WP-sensitized animals, we examined the tryptase and occludin gene expression in the ileum (Fig. 6). Tryptase is a protease present in mast cell granules and it has been shown to decrease the level of a tight junction protein, occludin [32]. Although we did not observe TB-stained mast cells in the intestines from the young mice (Fig. 5a), we found that tryptase expression (*Tpsab1*) was significantly increased in the young male mice. In agreement with this observation, the expression of occludin (*Ocln*) was decreased, suggesting that increased levels of tryptase might have affected the integrity of tight junctions by decreasing occludin expression in these mice. To our surprise, *Ocln* expression was increased in WP-sensitized young female mice while their *Tpsab1* expression was unaffected. Although we do not have an explanation for this phenomenon at this time, an increased expression of another tight junction protein, claudin-2, in association with mast cell activation, has been reported in irritable bowel syndrome patients [61]. Nevertheless, the decreased occludin expression observed in the young male mouse intestine suggested that the normal feature of the ileum was disrupted and therefore intestinal barriers were compromised. Such “leaky gut” likely allows inappropriate entry of food stuff and intestinal bacteria into intestinal walls, leading to further inflammatory events. Because inflammatory factors such as interleukin (IL)-4, IL-5, IL-13, IL-15, eotaxin-3 [62, 63], IL-10, tumor necrosis factor (TNF)-α, and interferon (IFN)-γ [64], are found to be elevated in non-IgE-mediated food allergy, it is also our future interest to examine the changes in these inflammatory factors in our mouse model.

In contrast to the age-specific pattern of mast cell numbers we observed in the ileum, we found differences in the number of brain mast cells between young and older mice. Mast cells were present in both age groups and the majority were degranulated (Figs. 8 and 9), which may be physiological [17, 33] or have been triggered by stress during handling [34]. Although we were not able to directly compare the absolute number of brain mast cells in the young and old mouse brains due to differences in the number of tissue sections, a greater number of mast cells, especially degranulated mast cells, were present in the WP-sensitized male brains compared to the age-matched sham males or females in the young groups (Fig. 8). On the other hand, mast cells were only sporadically found in the brains of the old mice (Fig. 9). Because mast cell precursors are capable of migrating into the brain from the circulation [15–17], it is possible that the increased number of mast cells found in the WP-sensitized young mice may be the result of mast cell recruitment from the periphery. Brain mast cells were predominantly found in the subarachnoid space between the medial hippocampus and lateral midbrain (Fig. 7a), where one of the major cerebral arteries, the posterior cerebral artery (PCA) is positioned [65]. The PCA, along with the anterior and middle cerebral arteries, may therefore serve as the entry point for mast cells into the brain from the peripheral circulation. Similar distributions of mast cells in the brain have been reported [16]. Increased presence of IgE has been demonstrated in the brain of ovalbumin-sensitized mice [18], thus it is possible that these brain mast cells become associated with IgE and activated upon antigen challenge. In a study using casein as a food allergen, it was shown that serum casein level increased in orally sensitized mice after food challenge, indicating that food antigen is capable of entering into the circulation in a similar milk-allergy model [41].

In addition to the increased number of mast cells, the changes in 5-hmC staining patterns also verified more directly that WP sensitization influenced brain physiology. Although 5-hmC staining does not show which genes are undergoing epigenetic modification, it approximates the areas of the brain where such modifications are present. While we observed changes in the staining patterns in parts of the cerebral cortex and the thalamus, the most consistent changes were observed in the amygdala with increased staining in WP-sensitized mice. This observation suggested that the expression of certain genes in these brain regions become epigenetically regulated. Because these regions are important for motor, sensory, and emotional controls, it is reasonable to postulate that the modified genes take part in restructuring brain biochemistry and/or architecture to ultimately affect behavior. Identification of such modified genes with 5-hmC DNA immunoprecipitation and sequencing of the precipitated DNA may lead to further understanding in the pathophysiology of allergy-mediated behavior alteration.

As additional evidence for the influence of WP sensitization on the brain, we also examined whether microglia were activated. When overall brain microglial population was visualized with Iba1 staining, we found subtle, but notable, staining differences in the hippocampal regions of the WP-sensitized old male mice, indicating that microgliosis might be present in these animals (Fig. 11). In addition, there was profound hypertrophy of GFAP-positive astrocytes around the vasculature in the old WP-sensitized male brains (Fig. 12), suggesting that perivascular changes had occurred. Given the functions of the perivascular astrocytes in the control of cerebral blood flow and blood-brain barrier transport, [66–68], it is likely that WP-sensitization-induced phenotypic changes in the astrocytes altered these functions. Although astrocyte hypertrophy is often associated with disease states including depression [69], it is not clear whether it is a process toward pathology or a countermeasure. Indeed, astrocyte hypertrophy has been described as beneficial, at least acutely, for preventing synaptic loss in neuronal injury [70]. It is therefore plausible that the astrocytic hypertrophy observed in our WP-sensitized mice is a result of homeostatic effort to control leukocyte infiltration and/or cytokine influx during the allergy-mediated inflammatory state. Nevertheless, the blood-brain barrier integrity should be further assessed in future work to test this hypothesis as the morphological change could clearly reflect increased permeability of the blood-brain barrier as well.

A potential explanation for the lack of differences in brain mast cells in the old mouse groups may be that we did not detect all types of mast cells in the tissues with the acidic TB staining. Identification of mast cells by acidic TB staining relies on the property of mast cell granules as well as on tissue preparation methods [71]. Because mast cells are known to exist in multiple subtypes during different developmental and activation stages [72], other staining methodology, such as tryptase- or chymase-immunohistochemistry [71], should be considered to assure inclusion of all mast cell subtypes in our future analyses. This notion may also explain the discrepancy in the mast cell staining and the *Tpsab1* expression we observed in the ileum from the young WP mice. The *Tpsab1* expression we detected might have originated from different subtypes of mast cells that were not detected by TB staining.

Mast cells have been linked to neuropsychiatric symptoms. Patients with mastocytosis, a condition in which abnormal accumulation and/or degranulation of mast cells occur in various tissues, suffer from depression, anxiety, memory loss, attention and concentration deficits, poor motivation, and cognitive impairment, in addition to gastrointestinal and cardiovascular problems [73–75]. Although mastocytosis is a genetic disease [76], mast cells are also known to accumulate at the site of inflammation in various conditions such as ischemic or traumatic brain injuries [77, 78], parasitic infections [79], glioma [80], and multiple sclerosis [81]. It is therefore feasible that WP-sensitization-induced inflammation recruited mast cells into the brain and gut in our young and old male mice, respectively, which ultimately led to altered digging behavior. Digging behavior of mice is an instinctive survival activity to search for food, store food, and form dwellings [27]. A significant decrease in this activity may therefore signify their depressive state with lack of motivation to thrive. However, additional behavioral analyses are required to assess other neuropsychological aspects such as cognition and memory.

## Conclusions

While mast cells have been implicated in disorders of the CNS by a growing number of studies [82], potential roles of mast cells after a peripheral allergic challenge have not been explored. Our results demonstrated altered distributions of mast cells associated with behavioral abnormality in an age- and sex-dependent manner and presented supporting evidence for the involvement of mast cells in food allergy-induced behavioral problems. In addition to the vagus nerve and circulating proinflammatory cytokines, mast cells, therefore, provide an additional contributing mechanism for peripheral-to-central communications. Validating a causal role of food allergy in neuropsychiatric conditions will provide safe and inexpensive therapeutic approaches to control behavior abnormality with implementation of allergy tests and avoidance of offensive food items. Such preventative management may also ultimately decrease the use of behavior modifying medications and further reduce the risk of adverse side effects and costs.

## Abbreviations

ADHD: Attention-deficit hyperactivity disorder
CNS: Central nervous system
CT: Cholera toxin
ELISA: Enzyme-linked immunosorbent assay
GFAP: Glial fibrillary acidic protein
IgE: Immunoglobulin E
IL: Interleukin
INF-γ: Interferon-gamma
PBS: Phosphate-buffered saline
PBST: Phosphate-buffered saline with Tween-20
PCA: Posterior cerebral artery
TB: Toluidine blue
TNF-α: Tumor necrosis factor-alpha
WP: Whey proteins
